# 14.5 GHz passive harmonic mode-locking in a dispersion compensated Tm-doped fiber laser

**DOI:** 10.1038/s41598-017-06326-5

**Published:** 2017-08-10

**Authors:** Yazhou Wang, Jianfeng Li, Kundong Mo, Yanyan Wang, Fei Liu, Yong Liu

**Affiliations:** State Key Laboratory of Electronic Thin Films and Integrated Devices, School of Optoelectronic Information, University of Electronic Science and Technology of China, Sichuan, China

## Abstract

We demonstrate a high repetition rate passive harmonic mode-locking (HML) based on nonlinear polarization evolution (NPE) technique in a Tm-doped ring fiber laser cavity. Small net anomalous cavity dispersion based on dispersion compensation benefited the generation of high repetition rate HML due to the low soliton splitting threshold. Stable HML with a repetition rate of up to 14.5 GHz and a super-mode suppression (SSR) of 19 dB was obtained at the center wavelength of 1982.3 nm, which is about ten times of state of the art at 2 μm band mode-locking fiber laser to our best knowledge. The repetition rate was selectable between 1 GHz to 14.5 GHz through changing the pump power and intra-cavity polarization state, and the SSR better than 25 dB was obtained as the repetition rate less than 5 GHz.

## Introduction

Mode-locked fiber lasers operating at eye-safe 2 µm region have attracted wide attention due to its potential application in remote sensing^1^, medicine^2, 3^, LIDAR^4^, mid-IR generation^5–7^, and so on. In the past decades, a number of 2 µm mode-locked fiber lasers have been reported, however, there are few researches focused on the generation of high repetition rate pulses which is attractive for realizing high-speed optical communication, spectroscopy, and precision optical sampling. A direct method to achieve high repetition rate mode-locking is of improving the fundamental repetition rate by shortening the cavity length. Tm-doped mode-locked linear fiber lasers with fundamental repetition rate of 253 MHz^8^, 535 MHz^9^ and 982 MHz^10^ have been demonstrated by this way with a cavity length of ~40 cm, ~18.7 cm, and ~10 cm, respectively. Very recently, an 5.9 cm linear Tm-doped fiber cavity mode-locked by a semiconductor saturable absorber mirrors was proposed to increase the fundamental repetition rate to 1.6 GHz, which is the highest repetition rate around 2 µm as we best known^11^. However, further shortening of the cavity length will be unpractical due to the limitation of doping concentration of gain fiber and pump strength.

HML, caused by quantized multiple pulses formation per round trip due to soliton peak-power limiting effect, is an alternative scheme to multiply the pulse repetition rate to hundred even thousand times of the fundamental repetition rate without reducing the cavity length^12^. Several passive HML have been reported at 2 µm with NPE. HML with repetition rates of 537 MHz (14^th^) and 690 MHz (19^th^) has been observed in a Tm-doped NPR mode-locked fiber laser^13^. With similar scheme, pulse bundles and 15^th^ HML has been demonstrated in a long-cavity Tm-doped fiber laser, however, the maximum repetition rate was limited to 6.9 MHz due to the low fundamental repetition rate of 448.8 kHz. M. Pang *et al*. have demonstrated a 1.46 GHz (52^nd^) passive based on optoacoustic effects in a piece of nanobore photonic crystal fiber^14^. By combing NPR with a 80 cm Tm-doped linear fiber cavity, second order HML of 496 MHz has been reported in a short laser cavity of ~80 cm, in which a multifunctional hybrid fiberized device was applied to reduce the physical cavity length^15^. Real satuarable absorbers (SA) have also been served as a mode-locker to achieve HML. M. Zhang *et al*. have reported 387.6 MHz (60^th^) HML with graphene-polymer composite in a Tm-doped ring fiber laser^16^. By using carbon nanotube-based SA, 50 MHz (3^th^) and 213 MHz (25^th^) HMLs have been reported, respectively^17, 18^. 705.9 MHz (37^th^) HML based on fiber-taper-based black phosphorus has been observed in a Tm/Ho doped ring fiber laser^19^. A 70 cm Tm/Ho-doped linear fiber cavity combined with a semiconductor saturable Bragg reflector has also been proposed to obtain 270.4 MHz(2^nd^) and 405.6 MHz(3^th^) HML^20^.

A crucial factor for the limited repetition rates of above HML is the low harmonic order of less than 60^th^, and high order harmonic at hundred even thousand level still remained unexplored at 2 µm region. A valid way to generate high order harmonic is of reducing the soliton splitting threshold inside the laser cavity by reducing the net anomalous cavity dispersion or inducing high-nonlinear component, according to the soliton area theorem^14, 21, 22^. However, the mode-locked fiber lasers at 2 μm band generally operate at large anomalous dispersion regime as a result from the large anomalous dispersion of both SMF28e fiber and Tm-doped gain fiber, implying that the generation of high repetition rate HML will be possible if dispersion compensation is employed to reduce the large anomalous dispersion of the 2 µm laser cavity.

In this paper, a piece of normal dispersion fiber (DCF) with high nonlinear coefficient was used to achieve high repetition rate HML in a Tm-doped NPR mode-locked fiber laser. Stable HML with repetition rate up to 14.5 GHz and SSR of 19 dB was obtained, which is about 3436^th^ harmonic of the fundamental repetition rate. The repetition rate was selectable between 1 GHz to 14.5 GHz by adjusting the PCs and pump power, and the best SSR of 27 dB was obtained at 3.4 GHz.

## Results

**Figure 1:**
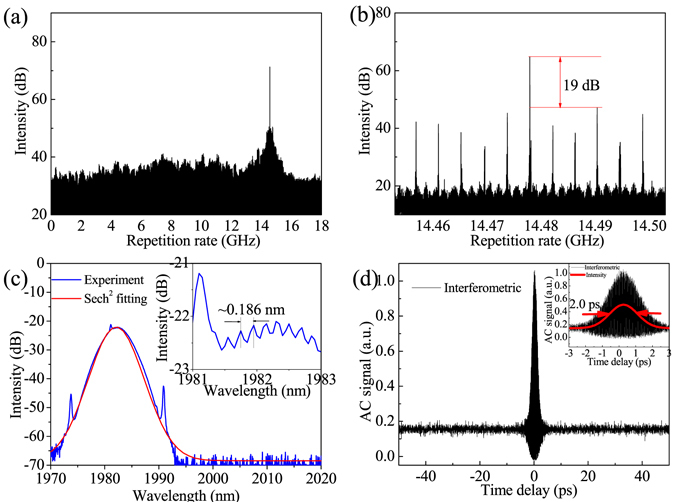
HML at repetition rate of 14.5 GHz: (**a**) and (**b**) are RF spectra with a scanning range of 18 GHZ and 50 MHz, respectively; (**c**) optical spectrum with 0.05 nm resolution, inset is a zoom of the spectrum; (**d**) interference autocorrelation with a scan range of 100 ps. Inset of (**d**) is interference and intensity autocorrelation with sech2-pulse fitting in a scan range of 6 ps.

The experimental setup of the high repetition rate passive HML as designed and constructed as described in the Methods part. The fundamental repetition rate of the mode-locked laser is 4.22 MHz. Initially, self-starting soliton bundles were formed at the pump power of 1.05 W. At the pump power of 2.4 W to 3.9 W, stable HML with selectable repetition rate of exceeding 1 GHz was obtained by adjusting the PCs. The highest repetition rate, obtained at the pump power to 3.2 W, was up to 14.5 GHz. The harmonic order was estimated to be 3436^th^ of the fundamental repetition rate, which is record-breaking at 1–2 μm spectral region as we best known. The measured output power of 9.3 mW corresponds to the soliton energy of ~0.64 pJ. Figure 1a shows the measured RF spectrum for a scanning range of 18 GHz with a resolution of 100 kHz. Only a single peak at 14.5 GHz was observed, indicating that the peak is the fundamental frequency component of the harmonic mode-locking instead of the repeatable high harmonic frequency component. High order radiofrequency components of the pulse train were not detected in Fig. 1a due to the limitation of the RF analyzer’s scanning range (18 GHz). Figure 1b shows the measured SSR of better than ~19 dB for a scanning range of 50 MHz with a resolution of 10 kHz. The SSR was further increased to the level of ~25 dB as the repetition rate less than 5 GHz. The uneven super-noise peaks with equal separation of 4.22 MHz is the typical feature of harmonic mode-locking, which caused by the correlations between pulses inside the cavity^23^. Note that the timing jitter as another characteristic of HML quality could not be directly measured from the RF spectrum due to the existence of super-mode noises^24^. Although the cross correlation is an alternative method^24^, the need of a delay range of hundreds picosecond level exceeds the scanning range of our optical correlator. Figure 1c shows the measured optical spectrum with a resolution of 0.05 nm. Typical Kelly sidebands caused by the interference between soliton and dispersive wave suggests the operation of conventional soliton mode-locking. The separation of 17.2 nm between first-order Kelly sidebands agrees well with the estimated net cavity dispersion of −0.21 ps^2^ according to the formula of $\Delta {\rm{\lambda }}=\frac{{\lambda }^{2}}{0.567\pi c\tau }\sqrt{-1+\frac{4\pi {(0.567\tau )}^{2}}{k^{\prime\prime} L}}$ ^25^, where τ donates pulse duration at half of the maximum intensity, $k^{\prime\prime}$ refers to the net cavity dispersion, and *L* is the cavity length. A self-generated CW component near the center wavelength was served as a long-range weak soliton interaction force to ensure the uniform distribution of pulses in the laser cavity^26, 27^. By fitting the spectrum with a sech^2^ shape, the spectral FWHM and center wavelength were estimated to be 3.77 nm and 1982.3 nm, respectively. Inset of Fig. 1c shows an in-zoomed spectrum from 1981 nm to 1983 nm, the spectral peaks with a separation of ~0.186 nm basically agrees with the repetition rate of 14.5 GHz, which might be caused by the modulation of very close pulses^28^. The spectral peak amplitude around center wavelength is only ~0.2 dB, and becomes weaker in the marginal region of the spectrum, which could be due to the limited resolution of 0.05 nm of our optical spectrum analyzer. Figure 1d shows the intensity autocorrelation trace with a scan range of 100 ps, where no additional pulses are observed, excluding the possibility of soliton bunches or soliton pairs. Inset of Fig. 1d shows the measured autocorrelation trace of the mode-locked pulses at a scanning range of 6 ps. The FWHM is 2.0 ps corresponding to the pulse duration of 1.3 ps if the sech^2^-pulse fit is assumed. The calculated time-bandwidth-product of 0.37 suggests that the soliton pulse was slightly chirped.

**Figure 2:**
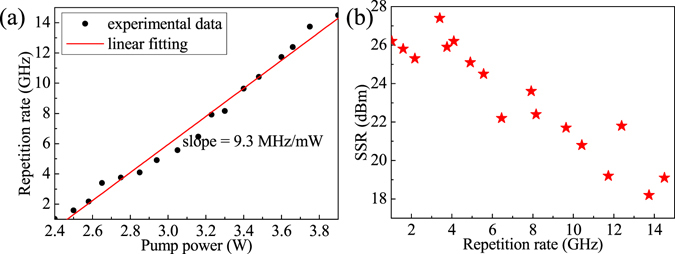
(**a**) Repetition rate of the HML as a function of pump power, and (**b**) SSRs of the HML at different repetition rates.

**Figure 3:**
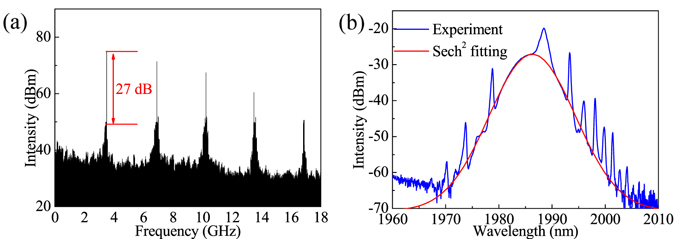
(**a**) RF spectrum of HML at repetition rate of 3.4 GHz with a scanning range of 18 GHz, and (**b**) Optical spectrum corresponds to (**a**).

Figure 2a shows the repetition rate of the HML as a function of pump power where the PCs’ positions were kept unchanged. The repetition rate near linearly increased from 1 GHz to 14.5 GHz with a slope of 9.3 MHz/mW as the pump power was increased from 2.4 W to 3.9 W. When the pump power further increased, the HML became unstable. Figure 2b shows the measured SSRs at different the repetition rates corresponding to Fig. 2a. The SSR is better than 25 dB when the repetition rate less than 5 GHz. The best SSR of 27 dB, as shown in Fig. 3a with a RF scanning range of 18 GHz and a resolution of 100 kHz, was obtained at the repetition rate of 3.4 GHz with pulse duration of 950 fs and soliton energy of ~1.89 pJ. Figure 3b shows the corresponding optical spectrum with a sech^2^-fitted FWHM of 6.2 nm and center wavelength of 1986.6 nm. Compared to the 14.5 GHz HML case, spectral modulation was not observed due to the estimated modulation period of ~0.04 nm cannot be resolved by our optical spectrum analyzer, and the center wavelength drifted to 1986.6 nm because of the adjustment of PC position. Additionally, stronger Kelly sidebands in this case were observed, and the wavelength separation between first-order Kelly sidebands decreased to 14.6 nm. These are the signs of higher soliton energy and narrower pulse duration^25^.

## Discussion

**Figure 4:**
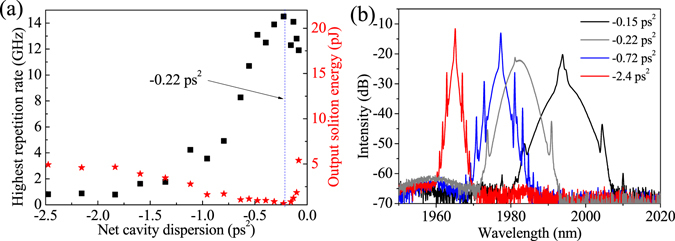
(**a**) Highest repetition rate of stable HML and corresponding soliton energy at different net cavity dispersions, and (**b**) optical spectra at the net dispersion of −0.15 ps^2^, −0.22 ps^2^, −0.72 ps^2^, and −2.4 ps^2^.

The DCF fiber played a crucial role for the achievement of high-order HML in our experiment. To validate that, we adjusted the net cavity dispersion by changing the length of SMF28e fiber from 19 to 49.5 m. Figure 4a shows the obtainable highest repetition rate of stable HML and corresponding soliton energy at different values of net cavity dispersion. The soliton energy basically reduced as the net dispersion varied from −2.5 ps^2^ to −0.22 ps^2^, while the repetition rate gradually increased to GHz level and finally higher than 10 GHz. As the net cavity dispersion further varied from −0.22 ps^2^ to −0.06 ps^2^, the repetition rate of the HML basically decreased due to the abnormally increased soliton energy induced by the high soliton energy feature of stretched soliton at near zero dispersion regime^29^, suggesting that the net cavity dispersion around −0.22 ps^2^ is approximately suitable for the generation of high repetition rate HML. Figure 4b shows three sample spectra at net dispersion of −0.15, −0.72, and −2.4 ps^2^, respectively. With increasing of the anomalous net cavity dispersion, both spectral FWHM and wavelength separation between first-order Kelly sidebands were becoming narrower, which is coincident with the results reported in refs 25 and 30.

Currently there are few researches about the roles of self-generated cw component in HML^26, 27^, and how cw component affects HML performance is nearly unexplored. In our experiment, the cw component showed different states at different cases. For instance, the cw components in 14.5 GHz and 3.4 GHz cases presented different amplitudes and widths, as shown in Figs 1c and 3b. The wavelength separation between cw and spectral peak also seemingly related with the harmonic performance. For HML at GHz level, the separation was always larger than ~1 nm. Once the cw wavelength overlapped with the soliton’s center wavelength, the order of HML would be limited to MHz level. All above phenomena imply that the HML performance relates to the cw component parameters including amplitude, width, and wavelength relative to the center wavelength of soliton pulse. Detail investigations on this relationship will be performed in our future work.

## Conclusions

In conclusion, we report a high repetition rate HML based on a Tm-doped fiber laser with NPR mode-locking. To increase the harmonic order, a 20.0 m DCF fiber with high nonlinear coefficient was used to reduce the soliton splitting threshold. Stable pulse train with GHz level repetition rate was achieved at 2 μm band. The attainable highest repetition rate is 14.5 GHz with measured SSR of 19 dB, corresponding to pulse duration of 1.3 ps and center wavelength of 1982.3 nm. The best SSR of 27 dB was obtained at repetition rate of 3.4 GHz with pulse duration of 950 fs.

## Methods

### Experimental setup

**Figure 5:**
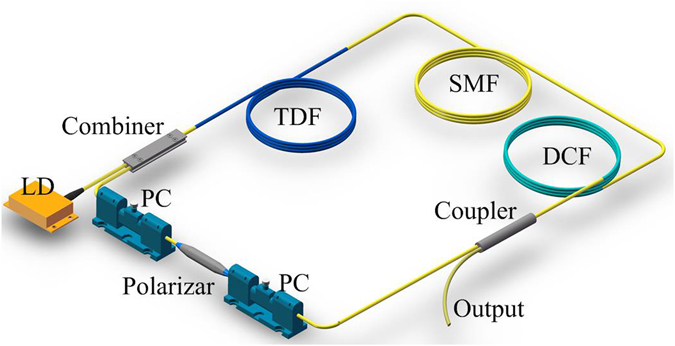
Setup of the mode-locked fiber laser.

Figure 5 shows the experimental setup. A 793 nm diode lasers (BWT) with a max output power of 12 W was lunched through a (2 + 1)×1 pump combiner (ITF, Canada) to 3.8 m double-clad Tm^3+^-doped fiber (Coractive, DCF-TM-10/128) with an octagonal shaped inner cladding with a diameter of 128 µm and a numerical aperture (NA) of 0.45. A 1/99 fiber coupler with only 1% output port was used to facilitate pulse splitting. A polarization dependent optical isolator with a polarization extinction ration of 35 dB at 2 µm (Advanced Photonics, USA) was used to induce NPE effect and ensure the unidirectional propagation. Two PCs were used to control the mode-locking performance. The total laser cavity length was about 44.8 m including 3.8 m Tm-doped fiber, 20 m DCF (OFS, LP980), and 21 m SMF 28e fiber. The dispersion values of the gain fiber and SMF28e fiber at 1.99 µm are ~−84 ps^2^/km and ~−80 ps^2^/km, respectively^31^. The dispersion value of DCF at 1.99 µm is ~−84 ps^2^/km, which was estimated in the mode-locked fiber laser with near zero net cavity dispersion. The nonlinear coefficients of these fibers are respectively estimated to be ~1.0 W^−1^km^−1^, ~8.6 W^−1^km^−1^, and ~0.9 W^−1^km^−1^. Therefore, the net second dispersion and average nonlinear of the cavity was thus respectively estimated to be ~−0.22 ps^2^ and ~2.43 W^−1^km^−1^.

### Measurement method

The output signal was detected by an InGaAs photodetector with bandwidth over 12.5 GHz (EOT ET-5000F, USA) was connected to a radiofrequency (RF) spectrum analyzer (CETC AV4033A, China). Interference autocorrelator (APE Pulsecheck USB 150, Germany) and optical spectrum analyzer (Yokogawa AQ6375, Japan) were used to measure the pulse duration and optical spectrum.
